# Similarity and Genetic Variation of Climbing Perch, *Anabas testudineus* (Bloch, 1792), From Java, Sumatra, and Kalimantan Islands, Indonesia

**DOI:** 10.1155/sci5/3009542

**Published:** 2025-09-02

**Authors:** Rudhy Gustiano, Ulfa Fayumi, Muhammad Hunaina Fariduddin Aththar, Irin Iriana Kusmini, Gadis Sri Haryani, Firman Muhammad Nur, Yosmaniar, Nurjirana, Umi Chodrijah, Amran Ronny Syam, Lukman, Titin Kurniasih, Safar Dody

**Affiliations:** ^1^Museum Zoologicum Bogoriense, Research Center for Biosystematics and Evolution, National Research and Innovation Agency, Cibinong 16911, West Java, Indonesia; ^2^Research Institute for Freshwater Aquaculture and Fisheries Extension, Bogor 16152, West Java, Indonesia; ^3^Research Center for Applied Zoology, National Research and Innovation Agency, Cibinong 16911, West Java, Indonesia; ^4^Research Center for Limnology and Water Resources, National Research and Innovation Agency, Cibinong 16915, West Java, Indonesia; ^5^Research Center for Marine and Land Bioindustry, National Research and Innovation Agency, Serpong, South Tangerang 15314, Indonesia; ^6^Graduate School, Hasanuddin University, Jl. Perintis Kemerdekaan Km 10, Tamalanrea, Makassar 90245, South Sulawesi, Indonesia; ^7^Research Center for Fisheries, National Research and Innovation Agency, Cibinong 16911, West Java, Indonesia; ^8^Research Center for Conservation of Marine and Inland Water Resources, National Research and Innovation Agency, Cibinong 16915, West Java, Indonesia; ^9^Research Center for Oceanography, National Research and Innovation Agency, Serpong, South Tangerang 15314, Indonesia

**Keywords:** *Anabas*, climbing perch, genetic variance, morphometric, RAPD

## Abstract

Climbing perch, *Anabas testudineus*, is an economically important freshwater fish in Indonesia. The climbing perch, also known as betok, has emerged as a prominent species in aquaculture due to its air-breathing ability, which allows the climbing perch to thrive in low dissolved oxygen environments. However, there is a lack of information on the genetic diversity of climbing perch from potential sources of populations as candidates for sustainable culture development. This study aimed to analyze the similarity, genetic distance, and diversity of climbing perch from Java, Sumatra, and Kalimantan Islands. We examined 21 truss morphometric characters to determine the intrapopulation variation. In addition, to assess genetic diversity and phylogenetic relationship between populations of climbing perch, we used random amplified polymorphic DNA (RAPD) with primers OPA 07, OPC 02, and OPC 05. The result showed that the population of climbing perch from Kalimantan shows higher similarity with the population from Sumatera (49.97%) than the population from Java (24.96%). Climbing perch from Kalimantan showed the highest polymorphism and heterozygosity of 39.29% and 0.16%, respectively. The interpopulation genetic distance between Kalimantan, Sumatera, and Java ranged from 0.17 to 0.39. The result suggests that the climbing perch from Kalimantan has potential as a candidate for the culture development of the climbing perch.

## 1. Introduction

Climbing perch *Anabas testudineus* (Bloch, 1792) is categorized in the Anabantidae family. The climbing perch was distributed from India to the Wallace Line, including China, and may have been distributed in more areas than were commonly reported [1]. Climbing perch, *A*. *testudineus*, is an economically important freshwater fish and emerged as a prominent species in aquaculture in Indonesia [2] and in South Asia [3, 4]. In Indonesia, climbing perch can be found in rivers, swamps, ponds, and estuaries. Furthermore, due to its unique ability of air breathing using the additional respiratory organ [5], the climbing perch can thrive in an environment with low water quality conditions, turbid, and stagnant water. During the rainy season, the climbing perch migrates from the river to the flood swamp area and returns to the river after the dry season. The climbing perch prefers areas abundant with aquatic plants [6] or with suspended plants [7].

Knowledge about existing morphological, physiological (production profile), and genetic diversity of any animal resource is an essential prerequisite to establishing effective utilization and conservation programs [8]. The wild climbing perch was massively exploited in several locations in Indonesia including South Sumatra [9], in Kalimantan [10, 11], and South Sulawesi Province [12]. The recent IUCN status of *A*. *testudineus* is least concern. Meanwhile, the population characteristics and the level of exploitation of climbing perches in India have been taken into account [13]. However, for further sustainable utilization, the data and information on the diversity of several candidate populations in Indonesia are needed.

The diversity within a population can be analyzed using various morphometric characters and genetic data [14–16]. The higher levels of diversity enhance the fitness of a population in terms of survival and adaptation [17–19]. Previous studies showed that morphometric analysis can differ in the population of climbing perch, including conventional [20], truss network [20–22], and geometric analysis [23, 24]. These methods effectively differ in variety and population from different environments. The genetic analysis using random amplified polymorphic DNA (RAPD) showed different banding patterns between populations of climbing perch [25], while mitochondrial analyses can separate clusters according to the genetic variance/population of climbing perch geographical location [23, 26] and showed a low genetic variation but with high population differentiation [27]. The morphometric and genetic analyses can analyze the diversity between populations to find the potential population with high genetic diversity for selective breeding. This study aimed to analyze the similarity, genetic distance, and diversity of climbing perch from Java, Sumatra, and Kalimantan. The candidate population for further aquaculture development of climbing perch in Indonesia was expected to be determined using the results from this study.

## 2. Materials and Methods

**Figure 1:**
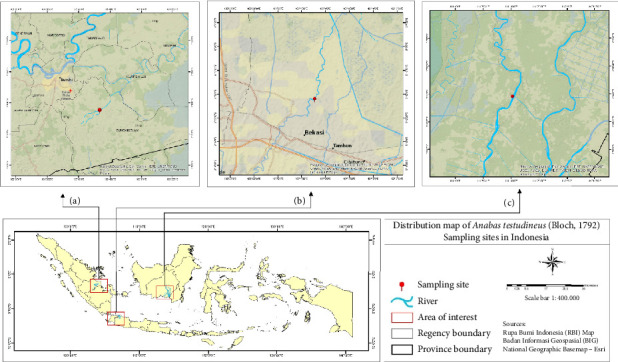
Location samples of *Anabas testudineus*: Sungai Gelam in Sumatra (a); Bekasi in Java (b); Pulang Pisau in Kalimantan (c).

We collected samples from three different locations including Sungai Gelam, Jambi Province in Sumatra Island (1°34′16.69 S 10°34′16.69″ E); Bekasi, West Java Province (−6°14′5.64″ S 106°59′22.56″ E) in Java Island; and Pulang Pisau, Central Kalimantan Province (2° 56′ 9.492″ S 115° 23′ 16.764″ E) in Kalimantan Island (Figure 1). We examined 30 fish samples per population for truss morphometric analysis and 10 samples per population for RAPD analysis.

### 2.1. Truss Morphometric

**Figure 2:**
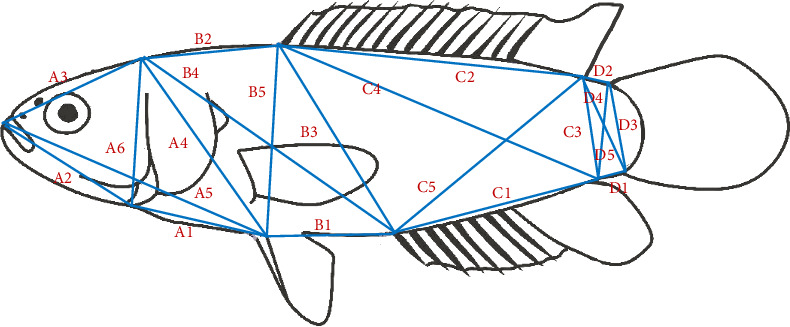
Truss morphometric characters on *Anabas testudineus*.

We examined 21 truss morphometric characters per fish (Figure 2; Table 1). We used a digital caliper with a level of accuracy of 0.01 mm. Measurements were made using digital calipers with an accuracy level of 0.01 mm. All character morphometric measurements were divided by standard length. Measurements were then log-transformed in order to minimize the effect of non-normality. Data were subjected to canonical discriminant function analysis (DFA). Finally, data analysis consisted of characterizing groups from scatter plots using DFA.

### 2.2. Genetic Analysis

We prepared the samples using the Tiagen DP324 product, a marine animal DNA kit from Magen Biotechnology Company Ltd., PRC. A 1.5 mL microcentrifuge tube was used for the 30 mg fin clip samples. For PCR–RAPD, we used the primers OPA 07, OPC 02, and OPA 05. We conducted the amplification process in a total reaction volume of 19 μL, containing 3 μL DNA, 1 μL primer, 10 μL Taq DNA polymerase (MyTaq HS Mix Bioline PCR BIO-25045, Meridian Life Science Inc., USA), and 6 μL Aquades. Then, we set the PCR condition as follows: 1 denaturation cycle at 94°C for 5 min, followed by 40 doubling cycles, consisting of denaturation at 94°C for 40 s, annealing at 35°C for 1 min, and extension at 72°C for 2.5 min ending with a final extension at 72°C for 7 min. 10 μL of PCR products [28] was then electrophoresed using 2% agarose gel in 1% buffered Tris-Boric EDTA (TBE). We observed and processed the electrophoresis result using a Geldoc (Vilber Lourmat E-BOX VX5 20 M Products). To examine genetic diversity, we used Tools for Population Genetic Analysis (TFPGA); (Nei and Tajima 1981 [29]). Finally, we visualized the interpopulation kinship relationship using a dendrogram created by the unweighted pair genetic method with arithmetic mean (UPGMA).

## 3. Results and Discussion

### 3.1. Morphometric Characteristics

**Figure 3:**
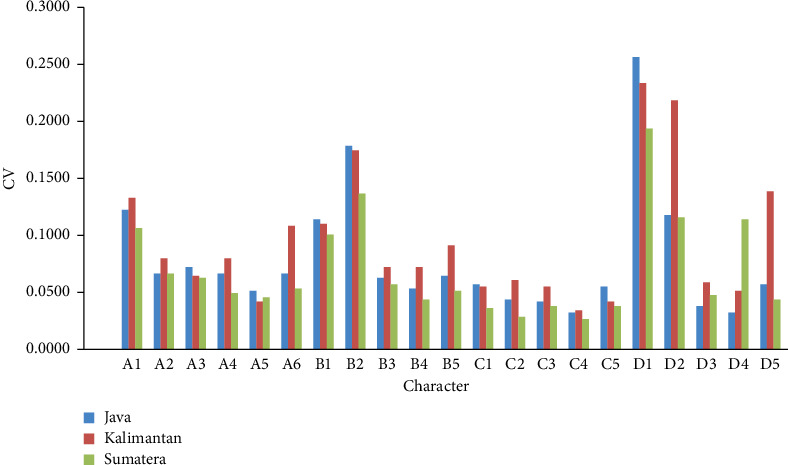
Coefficient of variation (CV) for truss morphometric character of three populations of *Anabas testudineus* (Java, Kalimantan, and Sumatera).

The morphometric analysis showed that A6 (the distance between the skull tip and the lower operculum), C1 (the distance between the start of the anal fin and the end of the anal fin), and C3 (the distance between the end of the dorsal fin and the end of the anal fin) were significantly different (*p* ≤ 0.05) between populations (Table 2). The morphometric characters exhibited a coefficient of variation (CV) ranging from 0.03 to 0.27 (Figure 3). The Character D1 (the distance between the end of the anal fin and the start of the lower caudal fin) had the highest CV, ranging from 0.2 to 0.27. In contrast, C4 (the distance between the start of the dorsal fin and the end of the anal fin) had the lowest CV at 0.03 (Figure 3).

**Figure 4:**
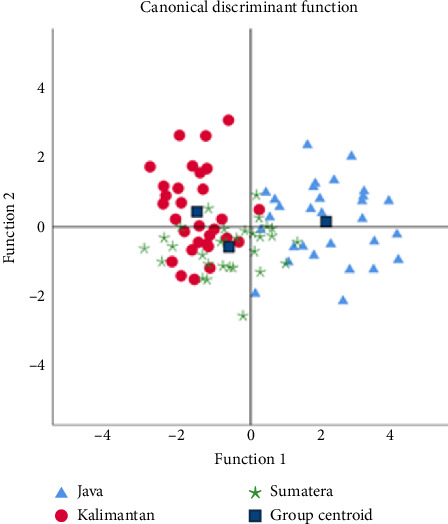
Plot of Function 1 versus Function 2 taken on a DFA of analysis on 21 log-transformed metric variables on 30 samples of *Anabas testudineus*.

**Figure 5:**
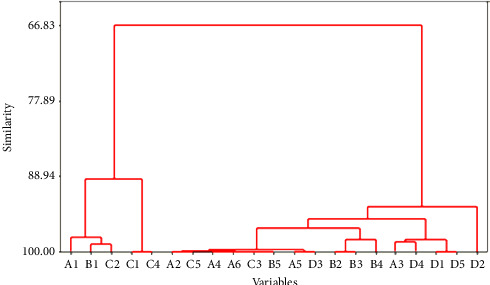
The similarity of 21 truss morphometric characters for intrapopulation correlation of *Anabas testudineus*.

**Figure 6:**
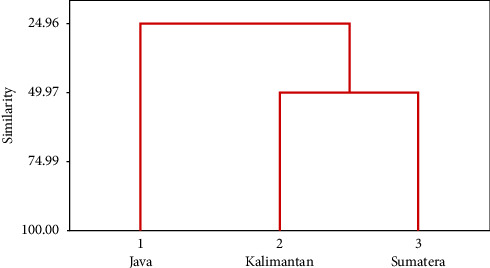
Dendrogram for the similarity of three populations of *Anabas testudineus*.

Scatter plot DFA showed that Function 1 successfully separated the samples into two groups; the Java population was distinctly separated, while the Kalimantan and Sumatra populations were close together and overlapped (Figure 4). Based on the dendrogram analysis of the similarity of 21 truss morphometric characters (Figure 5), there were two character clusters at similarity levels of more than 90% and 66%, representing the separation of Group 1 (Character A1, the distance between the beginning of the pelvic fin and the bottom of the operculum) and Group 2 (Character D2, the distance between the end of the dorsal fin and the beginning of the upper caudal fin). The dendrogram of interpopulation relationships of climbing perch indicated that the Sumatra population was 49.97% like the Kalimantan population, forming one cluster, and was separated from the Java population with a similarity level of 24.96% (Figure 6).

### 3.2. Genetic Analysis

**Figure 7:**
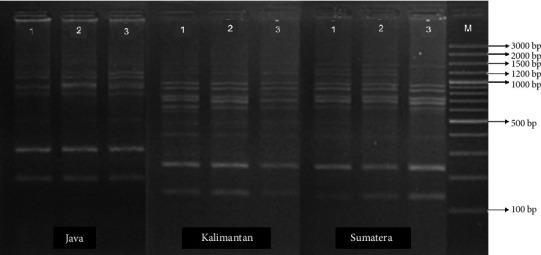
Mitochondrial DNA amplification using the primer OPA 07 on the populations of *Anabas testudineus* from Java, Kalimantan, and Sumatra Islands, Indonesia.

**Figure 8:**
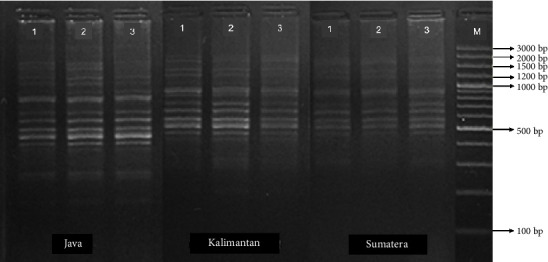
Mitochondrial DNA amplification using the primer OPC 02 on the populations of *Anabas testudineus* from Java, Kalimantan, and Sumatra Islands, Indonesia.

**Figure 9:**
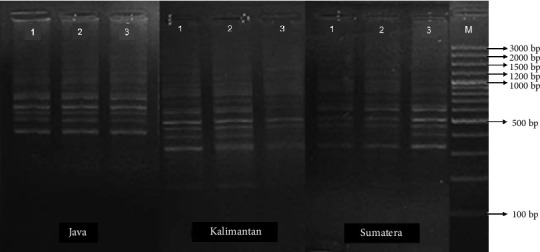
Mitochondrial DNA amplification using the primer OPC 05 on the populations of *Anabas testudineus* from Java, Kalimantan, and Sumatra Islands, Indonesia.

The DNA amplification results of the three populations of climbing perch (Figures 7, 8, and 9) revealed fragments of varying sizes between 7 and 15, within a range of 175–1750 bp. The highest number of fragments was found in the Kalimantan population, which had 12–15 fragments with sizes ranging from 175 to 1750 bp. In contrast, the Sumatra and Java populations exhibited 7–12 fragments, with sizes ranging from 175 to 1750 bp for the Java population and from 175 to 1500 bp for the Sumatra population.

The highest percentage of polymorphism was found in the Kalimantan population, at 39.29%. The percentages of polymorphism for the Java and Sumatra populations were equal, each at 28.58%. The heterozygosity values for the climbing perch across the three populations ranged from 0.09 to 0.16, with the highest heterozygosity in the Kalimantan population (0.16) and the lowest in the Sumatra population (0.09).

**Figure 10:**
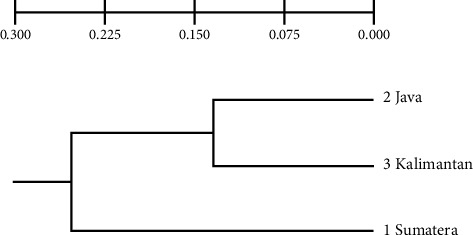
Genetic relationship of the populations of *Anabas testudineus* from Java, Kalimantan, and Sumatra Islands, Indonesia.

The FST pairwise test indicated a significant difference in genetic variance between the three populations of climbing perch (*p* ≤ 0.05). The Sumatra population had the greatest genetic distance from the Kalimantan population (0.39), while the genetic distance between the Java and Kalimantan populations (0.17) was closer than that between the Java and Sumatra populations (0.29). The Java population of climbing perch formed a single cluster with the Kalimantan population, whereas the Sumatra population was separated from both the Kalimantan and Java populations (Figure 10).

Morphological change can occur in 60 days [30]; meanwhile, another study [31] showed that the change between generations in a population can occur after 10–20 years. Fish morphology easily adapts due to the habitat, predation, food availability, and water current [32]. The environmental conditions are varied, and organisms handle this variability with two methods: local adaptation and phenotypic plasticity, with both tactics overlapping and interacting with each other.

In this study, three populations from different geographical locations can be distinguished using the phenotypic features of A6, C1, and C3. These three populations in the study are from different islands and showed small differences in phenotypic character. The results were similar for climbing perch populations from various geographical distributions in Malaysia, with only two significant features from the skeleton network [22], as well as the study of ten wetland populations, with only one population distinguishing itself from the other nine with a 38% similarity [21]. The morphometric network provides a higher total variance compared to conventional measurement [20]. This study demonstrated that the effect of local adaptation, phenotypic plasticity, and interaction with phenotypic variance in climbing perch populations from surrounding habitats resulted in relatively minor biological differences. The morphological differences were also found for adult males and females [33, 34]. Intraspecific phenotypic differences in climbing perch populations may be linked to habitat adaptations [35, 36]. Morphology has proven to be an effective method for assessing biodiversity and phylogenetic relationships between species with low levels of genetic diversity [37].

Wild populations of *Poecilia mexicana* were compared with fish reared in public garden habitats to investigate how genetic differences between populations and plasticity contribute to the phenotypic variation observed in nature [38]. They found evidence that genetic differences between populations influenced the expression of most traits, either independently or in combination with phenotypic plasticity and other predictive variables. Overall, their findings suggest that phenotypic differences between populations are driven, at least in part, by evolutionary changes rather than solely by flexibility resulting from environmental variation between habitats. Future studies should thoroughly assess whether evolutionary divergence is driven by natural selection and whether the traits observed represent adaptations to different ecological conditions.

Genetic analyses in this study showed that the three observed populations differed significantly in the number of fragments, polymorphisms, and heterozygosity, but these differences were not strongly reflected in morphology. In a similar study [25], it reported an average polymorphism rate of 69.29% in the populations they observed, which was higher than the values obtained in this study. It was found that five out of six populations of climbing perch from Sumatra and the Malay Peninsula exhibited no genetic variation in the mtDNA control region [27]. Thus, it is possible that the relatively lower genetic variation in the observed climbing perch populations was insufficient to produce significant morphological differences. In this study, the results indicated that the populations from Kalimantan and Sumatra were more closely related compared to the population from Java based on morphometric characters (Figure 3). However, genetically, Kalimantan and Java were more closely related than either was to Sumatra (Figure 9). This study also showed that, genetically, the Kalimantan population exhibited the highest diversity and polymorphism/heterozygosity compared to the other populations. It is possible that the high levels of diversity and polymorphism/heterozygosity contributed to the genetic similarity and relatedness of Kalimantan to the other two populations.

Widespread species often face substantial environmental gradients across their distribution range. For widespread and live-bearing fish such as the sailfin molly (*Poecilia latipinna*), genetic, life history, and environmental data have been used to evaluate the structuring of their populations [39]. Using microsatellite DNA, genotypes were generated for 168 individuals from 18 groups spanning most of the distribution range of the sailfin molly. They identified six distinct genetic groups using microsatellite data, with significant evidence of isolation by distance. They also observed a considerable number of migrants between neighboring communities. Despite the genetic structure, no evidence of cryptic speciation was found. The genetic clustering and migration patterns did not correspond to paleo drainage patterns. Life history traits varied among populations but were not easily interpretable.

Phenotypic plasticity, also known as environmentally influenced phenotypic variation, is the ability of a genotype to produce a range of phenotypes under different environmental conditions [40]. When analyzing a trait, it is crucial to consider the genetic architecture and environmental sources of phenotypic variation, as environmental changes can influence the genetic structure and plasticity of a trait. Plasticity, which facilitates more rapid genetic adaptation, plays a vital role in helping organisms cope with rapidly changing environments. Transgenerational plasticity (TGP) is the ability of parents to influence the phenotype of their offspring without causing genetic changes in the offspring's genes. Environmental experiences of the parental generation and/or preceding generations can affect the genotype through mechanisms recognized as TGP. During these phenotypic changes, offspring may prepare their physiology to better align with future environmental conditions. Parents can adjust their offspring's phenotype based on environmental experiences from their own or previous generations without inducing genetic changes. These phenotypic shifts prepare the offspring to adapt their physiology to the environment. TGP allows real-time compensatory responses to environmental variations, which are predicted to enhance species resilience in the face of climate change, as such changes often occur faster than natural selection can act. TGP is now recognized as a system that enables organisms to respond more rapidly to environmental shifts. Several studies have demonstrated that parental exposure to environmental stressors can provide benefits to their offspring. Furthermore, TGP and immunological priming can be utilized to develop innovative techniques for producing resilient offspring. This has the potential to enhance resistance to various diseases and abiotic stressors within and across generations, offering a wide range of applications in fisheries and aquaculture.

Although substantial evidence exists regarding the influence of the environment on phenotypic variation, the fundamental mechanisms underlying this phenomenon remain poorly understood. Parental effects and epigenetic inheritance have been hypothesized as mechanisms supporting plasticity and transgenerational programming in offspring. Therefore, understanding the molecular processes through which organisms receive environmental signals and adjust gene expression to produce alternative phenotypes is crucial in the context of global climate change and disease outbreaks in aquaculture systems. Phenotypic plasticity supports acclimatization responses by shifting the average phenotype in a population, thereby mitigating the adverse impacts of global change. However, little is known about how phenotypic plasticity evolves across multiple generations [40].

In a study analyzing the stock structure of *Trichopodus trichopterus* from Indonesian waters using truss morphometry [41], habitat differences were found to significantly impact morphometric characteristics. Conversely, an ecophenotypic study on *Midas cichlid* fish [36] revealed morphometric changes in the *Midas cichlid* species from Lake Batur, driven by habitat differences and water quality. These differences were detected in the anterior and posterior body regions. Parameters such as temperature and the presence of aquatic vegetation, particularly *Azolla pinnata*, influenced fish distribution and body shape in Lake Batur. However, body shape could not be identified based on other parameters such as chlorophyll A, total nitrogen (TN), dissolved oxygen (DO), and total dissolved solids (TDSs). Future genetic studies could explain why fish groups with differing body types coexist in the same location.

In this study, the trait with the highest CV was D1 (the distance between the end of the anal fin and the base of the lower caudal fin). Although this trait did not show statistically significant differences (*p* ≥ 0.05), it indicated a high level of plasticity compared to 20 other morphometric traits. Biologically, the plasticity of an organism can result in high phenotypic diversity, as reflected in the CV. Organisms with high plasticity tend to exhibit greater variation in certain characteristics under different environmental conditions.

In evolutionary processes, plasticity can influence the CV within a population. If individuals with high phenotypic variation (plastic traits) are better able to survive and reproduce under varying conditions, the population's CV may increase. In natural resource management, understanding the plasticity of a species can aid in predicting population variation, measurable through the CV. This is critical for sustainable resource management. Thus, while plasticity and the CV are distinct concepts, they are interconnected in the context of adaptation and population variation within a system.

## 4. Conclusion

The Kalimantan population shows a higher similarity level with the Sumatra population (49.97%) compared to the Java population (24.96%). The level of polymorphism and heterozygosity in the Kalimantan *A. testudineus* fish population is the highest, with values of 39.29% and 0.16, respectively. The genetic distance between the Kalimantan, Sumatra, and Java *A. testudineus* fish populations ranges from 0.17 to 0.39. Based on these results, the Kalimantan *A. testudineus* fish population is recommended as a candidate population for the development of this species in aquaculture.

## Figures and Tables

**Table 1:** The description of 21 truss morphometric characters in *Anabas testudineus*.

No	Truss grid	Code	Description
1	Head	A1	The bottom part of the operculum to the origin of the ventral fin base
2		A2	The bottom part of the operculum to the tip of the upper mouth
3		A3	The tip of the upper mouth to the end of the cranial
4		A4	The end of the cranial to the origin of the ventral fin base
5		A5	The origin of the ventral fin base to the tip of the upper mouth
6		A6	The end of the cranial to the bottom part of the operculum

7	Anterior body	B1	The origin of the ventral fin base to the origin of the anal fin base
8		B2	The end of the cranial to the origin of the dorsal fin base
9		B3	The origin of the dorsal fin base to the origin of the anal fin base
10		B4	The origin of the anal fin base to the end of the cranial
11		B5	The origin of the dorsal fin base to the origin of the pelvic fin base

12	Posterior body	C1	The origin of the anal fin base to the end of the anal fin base
13		C2	The origin of the dorsal fin base to the end of the dorsal fin base
14		C3	The end of the dorsal fin base to the end of the anal fin base
15		C4	The origin of the dorsal fin base to the end of the anal fin base
16		C5	The end of the dorsal fin base to the origin of the anal fin base

17	Caudal peduncle	D1	The end of the anal fin base to the ventral anterior of the caudal fin
18		D2	The end of the dorsal fin base to the dorsal anterior of the caudal fin
19		D3	The dorsal anterior of the caudal fin to the ventral anterior of the caudal fin
20		D4	The end of the dorsal fin base to the ventral anterior of the caudal fin
21		D5	The end of the anal fin base to the dorsal anterior of the caudal fin

**Table 2:** The average of 21 morphometric characters of *Anabas testudineus* from Kalimantan, Sumatera, and Java.

Character	Java	Kalimantan	Sumatera	Levene's test
A1	0.55 ± 0.29	0.79 ± 0.40	0.66 ± 0.39	0.93
A2	1.05 ± 0.66	1.14 ± 0.67	0.92 ± 0.55	0.377
A3	1.00 ± 0.66	1.26 ± 0.76	1.09 ± 0.71	0.343
A4	1.26 ± 0.80	1.59 ± 0.96	1.18 ± 0.74	0.21
A5	1.51 ± 0.99	1.82 ± 1.11	1.51 ± 0.97	0.646
A6	1.11 ± 0.71	1.35 ± 0.81	1.02 ± 0.64	0.010∗
B1	0.76 ± 0.42	1.16 ± 0.62	0.88 ± 0.51	0.798
B2	0.43 ± 0.20	0.48 ± 0.23	0.40 ± 0.19	0.287
B3	1.46 ± 0.93	1.96 ± 1.17	1.47 ± 0.91	0.607
B4	1.67 ± 1.08	2.26 ± 1.36	1.68 ± 1.06	0.294
B5	1.21 ± 0.77	1.52 ± 0.90	1.14 ± 0.71	0.105
C1	1.33 ± 0.88	1.76 ± 1.09	1.40 ± 0.89	0.049∗
C2	2.16 ± 1.44	2.78 ± 1.71	2.27 ± 1.46	0.179
C3	0.64 ± 0.40	0.79 ± 0.47	0.63 ± 0.37	0.007∗
C4	2.37 ± 1.59	3.20 ± 1.98	2.49 ± 1.61	0.828
C5	1.52 ± 0.99	1.95 ± 1.20	1.56 ± 0.98	0.073
D1	0.28 ± 0.11	0.27 ± 0.09	0.24 ± 0.10	0.14
D2	0.30 ± 0.13	0.36 ± 0.13	0.31 ± 0.15	0.071
D3	0.59 ± 0.36	0.74 ± 0.42	0.59 ± 0.35	0.108
D4	0.65 ± 0.41	0.82 ± 0.48	0.67 ± 0.36	0.07
D5	0.65 ± 0.41	0.80 ± 0.44	0.66 ± 0.40	0.309

^∗^ significantly different (*p* ≤ 0.05).

## Data Availability

The data that support the findings of this study are available in the main text.
